# A Giant Cell Tumor of the Distal Femur Managed by Excision and Knee Arthrodesis Using a Custom Made Long Intramedullary Interlocking Nail: A Case Report and Review of the Literature

**DOI:** 10.7759/cureus.14810

**Published:** 2021-05-02

**Authors:** Anson Albert Macwan, Saurav N Nanda, Debashish Mishra, Saurabh Tuteja, Bodanapu Sandeep

**Affiliations:** 1 Orthopaedics, Kalinga Institute of Medical Sciences, Bhubaneswar, IND

**Keywords:** distal femur tumor, h3f3a gene, knee arthrodesis, long interlocking nail, megaprosthesis, neoplasm recurrence, giant cell tumor of bone, excision of tumor

## Abstract

Giant cell tumors (GCTs) are primary bone tumors that occur most commonly in long bones, with half such tumors occurring in the distal femur, proximal tibia, and fibula. Around 12% of patients present with a pathological fracture indicating more aggressive disease. Arthrodesis after tumor resection is a popular choice due to its affordability and early postoperative mobilization, as well as low risks of implant loosening, infections, malignant lesions, or mortality. A free fibular graft is a popular option in limb-sparing surgery for long bone tumors. A bone graft and nail can be used to reconstruct long bones and bridge defects up to 25 cm. In developing countries, the cost of the imported mega prosthesis, around 8,500 US$, means many patients cannot afford the treatment. We describe a case of a GCT of the distal femur treated by excision of the tumor and reconstruction using a fibular bone graft, with knee arthrodesis using a custom-made long intramedullary interlocking nail fixation across the femur to the knee and the tibia. The length was achieved with 1 cm shortening post-surgery. The result was satisfactory, and partial weight-bearing was allowed three months after the surgery. At the one-year follow-up, there was no recurrence, and the patient had the full weight-bearing ability.

## Introduction

A giant cell tumor (GCT) is defined as the presence of large multinucleated osteoclast-like giant cells, along with mononuclear spindle-like stromal cells and other monocytes [1]. The most common site of a GCT is the knee joint. Giant cells in GCTs lead to bone resorption [2]. Most cases of GCTs are associated with an *H3F3A* mutation, mainly *pG34W* [2]. GCTs account for 5% of all primary neoplasms and 20% of all benign bone tumors [3]. GCTs are most common among those aged 20-45 years [3]. They are slightly more common in females (ratio of 1.5:1) and relatively uncommon in young patients with an open physis [4]. The most usual presentation of a GCT is pain due to mechanical instability from bone resorption. A localized bony swelling may be observed due to destruction of the bone and tumor progression. GCTs are often found close to joints and hence cause a limited range of motion, joint effusion, and sometimes synovitis [4]. Around 12% of patients present with a pathological fracture, which indicates more aggressive disease, with the possibility of local recurrence post-treatment and metastasis in rare cases [5]. We describe a case of a GCT of the distal femur treated by an excision of the femur and reconstructed with a fibular bone graft and knee arthrodesis using a custom-made long intramedullary interlocking nail surgical technique. The goal of the treatment was to reduce the possibility of GCT recurrence and rarely metastasis post-surgery.

## Case presentation

A 29-year-old female came to the hospital with progressive swelling of the left distal femur associated with pain and swelling of the left knee for five months. There was no history of trauma, fever, weight loss, or any other abnormal swellings in the body.

**Figure 1:**
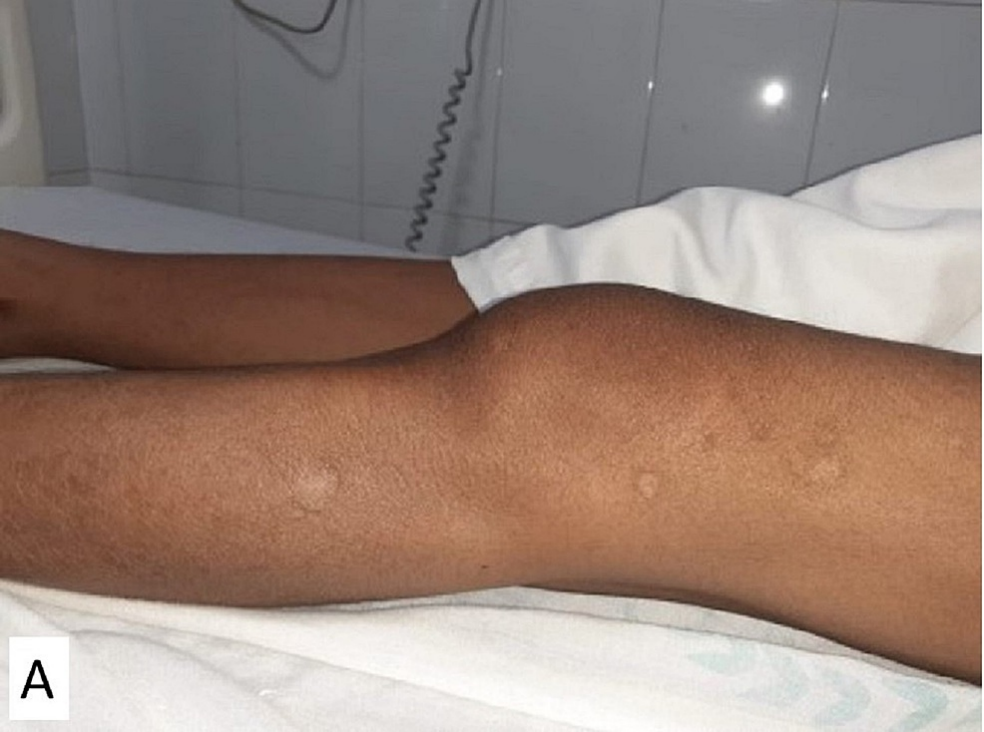
Clinical image of left distal thigh showing diffuse swelling

On examination, a single oval 7×5×2 cm swelling was seen over the lateral aspect of the left distal thigh (Figure 1). The skin overlying the swelling was normal in appearance, with no sinuses and no dilated or engorged veins.

**Figure 2:**
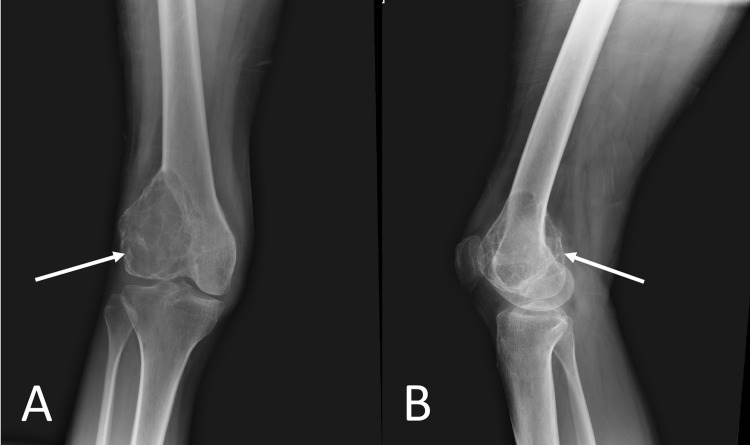
Plain radiograph of left knee joint Subarticular, eccentric, lytic lesion, with thinning of the cortex and no peripheral sclerosis in the lateral femoral condyle shown by the white arrow. (A) Anteroposterior view. (B) Lateral view.

All routine blood investigations and serum biochemistry studies were within normal limits. An x-ray of the left distal femur anteroposterior and lateral view showed a subarticular, eccentric, lytic lesion, with thinning of the cortex and no peripheral sclerosis in lateral femoral condyle (Figures 2A, 2B).

**Figure 3:**
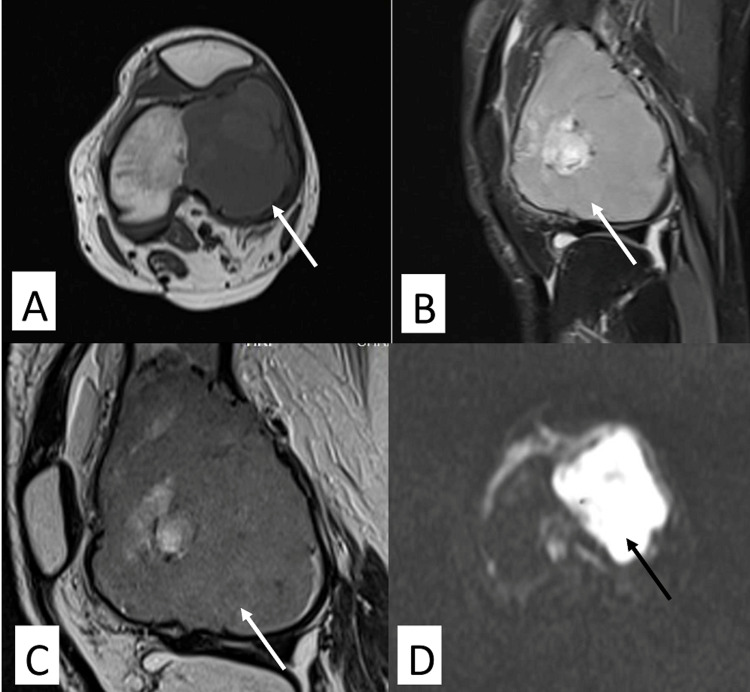
MRI images of the left knee joint showing lesion arising from the lateral femoral condyle (A) T1 axial section - lobulated hypointense lesion of the lateral femoral condyle (white arrow). (B) T2 sagittal section showing heterointense lobulated lesion (white arrow). (C) T2 thin sagittal section showing heterointense lobulated lesion (white arrow). (D) DIffusion weighted image showing diffusion restriction in the lateral femoral condyle (black arrow).

Magnetic resonance imaging of the left knee revealed an expansile lytic lesion (size: 6.6×4.2×5 cm) in the epi-metaphyseal region of the distal femur involving the lateral femoral condyle cortical breach and extending into the joint (Figures 3A-3D).

**Figure 4:**
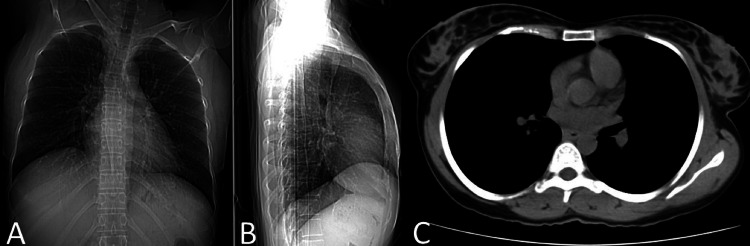
Chest radiograph and HRCT scan image showing no evidence of lung metastasis (A) Posteroanterior view of the chest radiograph. (B) Lateral view of the chest radiograph. (C) Axial section of HRCT. HRCT - High-resolution computed tomography

There was no evidence of lung metastasis on a chest x-ray and high-resolution computed tomography (Figures 4A-4C).

A core needle incisional biopsy was performed and showed tumor cells comprised of stromal cells containing diffuse giant osteoclast cells.

**Figure 5:**
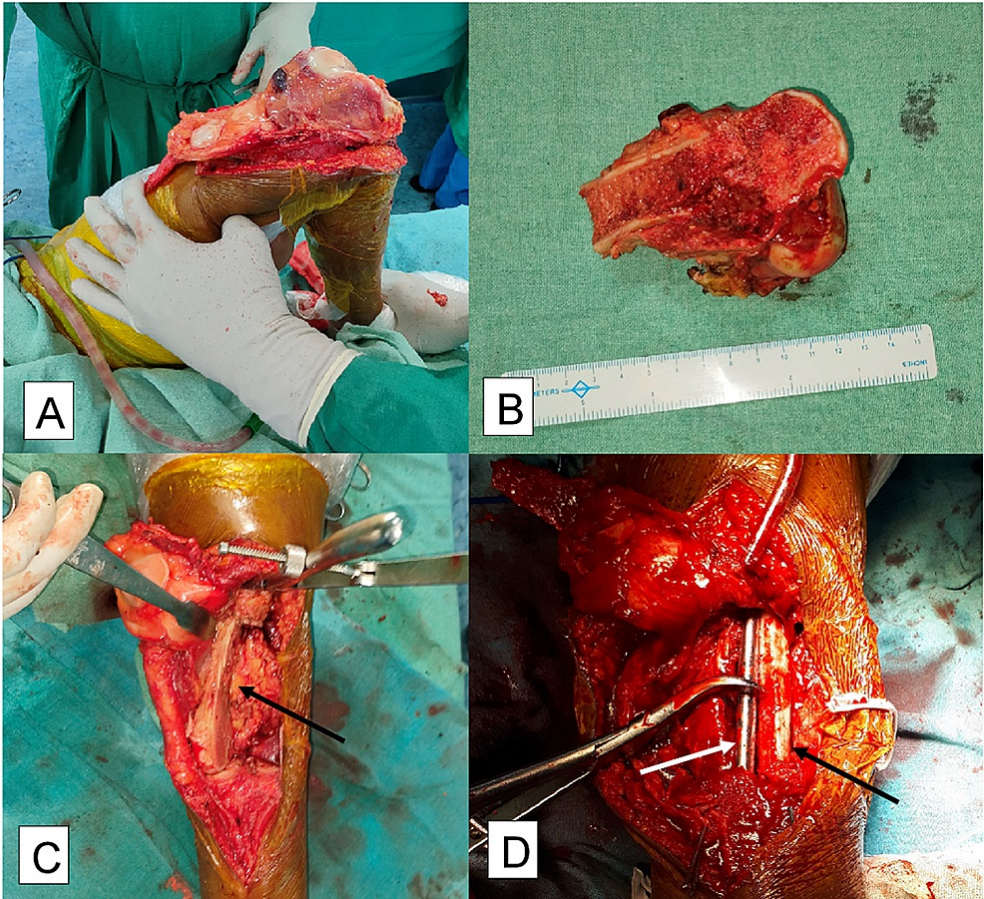
Intraoperative images (A) Left distal femur showing tumor. (B) Gross-excised tumor. (C) Defect after resection of tumor shown by the black arrow. (D) After reconstruction left arrow showing fibular strut graft and a white arrow showing part of locking intramedullary nail.

The tumour was approached by midline skin incision and lateral parapatellar approach. Tumour margins were identified and marked. A 9×5×4 cm area was excised from the left lateral femoral condyle, with a 2-cm clear margin. Both femoral and tibial cartilage surfaces were denuded and freshened to increase bony contact. Knee arthrodesis was performed using a custom-made long intramedullary interlocking nail (size 9×630 mm) fixed in both proximal femur and distal tibia. Ipsilateral fibular strut graft was used to fill the lateral distal femur void with 3.5 mm cortical screws. The posterior surface of patella was freshened and fixed to the tibia and femur using 4 mm cannulated cancellous screws (Figures 5A-5D).

Analysis of the biopsy sample confirmed a GCT. Adjacent soft tissue and articular cartilage and the proximal bony, intramedullary medial, and superior intramedullary margins were all tumor free. Necrosis was not present, with a mitotic count of 3/10 high-power field.

**Figure 6:**
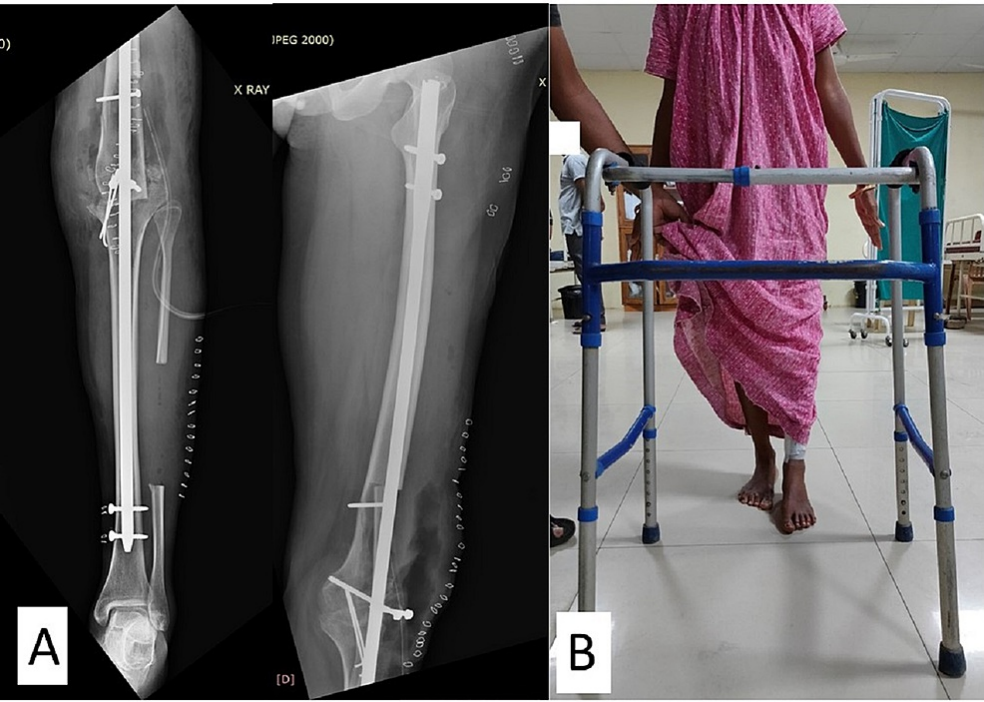
Postoperative radiograph and clinical image (A) Anteroposterior radiograph of left femur and tibia showing nail in situ. (B) Clinical image showing patient using a walker for mobilization.

**Figure 7:**
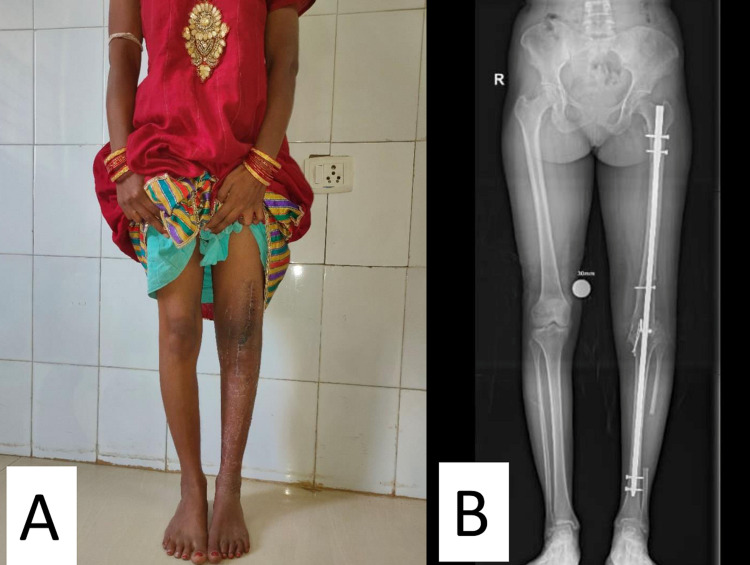
Clinical image and full length lower limb standing radiograph at one-year follow-up (A) Clinical image showing full weight-bearing. (B) Full-length lower limb radiograph showing long intramedullary locking nail in situ in the left lower limb with well callus formation at the arthrodesis site.

Postoperatively, the patient was stable with mild pain at the surgical site, which decreased gradually. The affected limb was 1 cm shorter than the healthy limb. The sutures were removed on the 14^th^ postoperative day, and the patient was allowed to mobilize, with non-weight bearing with the help of a standard walking frame (Figures 6A, 6B). Partial weight-bearing was allowed at three months after the surgery. There was no recurrence at the one-year follow-up, at which time the patient could walk, with full weight-bearing, without any support (Figures 7A, 7B).

## Discussion

GCTs, which commonly occur around the knee joint, are challenging to treat as the knee joint is the most crucial joint in the body in terms of weight-bearing. GCTs around knee joints commonly exhibit expansile growth and cortex thinning and commonly present as a pathological fracture [6,7]. Although GCTs close to the knee joint very rarely spread into the articular cavity, they still have adverse effects on knee function. Thus, preoperative planning is vital to reduce recurrence and preserve knee function in patients with GCTs [8].

A previous study compared recurrence rates in patients with GCTs after extended curettage and segmental resection around the knee joint [9]. In the extended curettage group (N = 69, 37 males and 32 females), there were five recurrence cases as compared with only one case in the segmental resection group (N = 24, 12 males and 12 females). In the same study, non-oncological complications were more common in the extended curettage group than in the segmental resection group. In another study, in cases of benign GCTs in the distal end of the femur where the articular margin of the knee was intact, the tumor was removed, and the cavity was eliminated by docking the bone fragments [10]. In this study, the knee joint and weight-bearing function of the limb were preserved. The patients in this study accepted limb shortening and had an excellent functional outcome. Persson and Rydholm described extensive local tumor excision in two GCT patients with joint fusion using a Kutscher nail and acrylic cement as a spacer [11]. Postoperatively, the weight-bearing ability was unaffected, with no complications, such as skin necrosis, nerve palsy, or infections. In the follow-up, three years in the case of the first patient and 16 months in the case of the second patient, there were no implant failures, recurrences, or fractures. In a review of patients with GCTs who were treated with extensive curettage with burr drilling and cryotherapy with liquid nitrogen, cryosurgery was successful in 100 of 102 patients [12]. Complications reported in this study included pathological fractures, skin necrosis, and significant degenerative changes [12]. Vicas et al. described the case of a 19-year-old male with a large, aggressive GCT treated with en bloc excision, a massive femoral allograft, and reconstruction of the ligaments [13]. In a follow-up of 18 years, there was no recurrence, with a good functional outcome. Natrajan et al. reviewed 143 patients with GCTs who underwent prosthetic arthroplasty, with a mean follow-up of five years [14]. They reported an excellent outcome in 62% of cases and a good outcome in 27% of patients using Enneking criteria [14]. Reported complications included periprosthetic fractures, infections, implant loosening, and recurrence. They concluded that good functional results can be achieved in selected cases of GCT, with fewer complications.

The optimum treatment for GCTs is en bloc resection. However, as shown in our case report, we obtained a good outcome in a patient with GCT with resection and reconstruction, with no recurrence up to one year post-surgery. Despite the availability of a variety of treatment options, long bone reconstruction after resection remains difficult. A free fibular graft is a popular option for limb-sparing surgeries in bone tumors [15,16]. In one study, 9 of 17 patients achieved a good outcome after autologous graft surgery, and in another study, 20 patients achieved a good outcome after autologous grafting in a 10-year follow-up both using Musculoskeletal Tumour Society functional score [17]. 

Autologous bone grafts and knee arthrodesis in GCT patients have long been used, and their effectiveness is well documented [18]. A bone graft and nail can be used to reconstruct long bones and bridge defects up to 25 cm. Arthrodesis after tumor resection is a popular option due to its affordability and early postoperative mobilization, as well as low risks of implant loosening, infections, malignant lesions, or mortality [18]. However, preoperative planning is vital to reduce recurrence and preserve knee function in patients with GCTs. Good outcomes in terms of the function of a limb can be achieved by filling the defect with an autograft, supported by a long intramedullary nail, with the bone graft fixed to the end of the host bone. By removing the need for a metallic implant, this method reduces postoperative complication rates.

The two most important determinants of surgery success in patients with GCTs of the femur are functional limb outcomes and tumor recurrence. As reported in the literature, there is a high recurrence rate after curettage and augmentation with a bone graft and a high recurrence rate after cryotherapy. However, en block excision of the tumor reduces the likelihood of recurrence to almost nil. Limb salvage is now possible due to the use of a mega prosthesis, which shows promising results in tumors requiring wide excision. However, the cost of the implant and the risk of implant loosening or failure are disadvantages of the mega prosthesis. Comparative outcomes have been achieved by artificial joint arthrodesis and an allograft [14].

Implants offer immediate stability and knee range of motion. However, in developing countries, the cost of imported implants, around 8,500 US$, means many patients cannot afford the treatment. Furthermore, implants have a finite lifetime. In India, affordable, high-quality implants are still not available [19]. Health care costs are a significant problem in India, as demonstrated in a previous study of 508 cancer patients in India [20]. In the study, 76% of the patients experienced financial difficulties due to cancer treatment costs. The surgical technique described in our report might help in treating aggressive tumors, such as GCTs, and improve the quality of life of the patient.

As science advances, there are new options, such as the mega prosthesis is available for GCTs affecting the knee. As reported in the literature, knee reconstruction after the excision of a large tumor is difficult. We excised the tumor with adequate margin and achieved good bone contact between the two segments. This was stabilized with lost cost custom-made long intramedullary locking nail. To further stabilize the construct, a fibular strut graft was put in the distal femur lateral side void. Using the surgical technique described herein, knee reconstruction can be achieved at a low cost.

## Conclusions

The optimum treatment of a GCT is surgery. Following resection of a tumor in the distal femur, knee reconstruction is difficult. With advances in medical science, new treatment options, including prosthetic joint replacement with a mega prosthesis, are available. However, the latter is associated with a risk of implant loosening and infections. It is also costly and requires a skilled surgeon to perform the surgery. Joint fusion using a long intramedullary nail is a cost-effective and alternative treatment option, which is easy to perform and allows early full weight-bearing postoperatively. This method also results in a pain-free functioning limb. Further research is needed on alternative methods for knee reconstruction after tumor resection.
